# Machine learning approaches that use clinical, laboratory, and electrocardiogram data enhance the prediction of obstructive coronary artery disease

**DOI:** 10.1038/s41598-023-39911-y

**Published:** 2023-08-03

**Authors:** Hyun-Gyu Lee, Sang-Don Park, Jang-Whan Bae, SungJoon Moon, Chai Young Jung, Mi-Sook Kim, Tae-Hun Kim, Won Kyung Lee

**Affiliations:** 1 School of Medicine, Inha University, Incheon, Korea; 2 Department of Cardiology, Inha University Hospital, School of Medicine, Inha University, Incheon, Korea; 3 Division of Cardiology, Department of Internal Medicine, Chungbuk National University College of Medicine, Cheongju, Korea; 4 ApexAI, Seongnam-Si, Korea; 5 Biomedical Research Institute, Inha University Hospital, Incheon, Korea; 6 Division of Clinical Epidemiology, Medical Research Collaborating Center, Biomedical Research Institution, Seoul National University Hospital, Seoul, Korea; 7 Department of Artificial Intelligence, Inha University, Incheon, Korea; 8 Department of Prevention and Management, Inha University Hospital, School of Medicine, Inha University, 27 Inhang-ro, Jung-gu, Incheon, Republic of Korea

**Keywords:** Cardiovascular diseases, Translational research

## Abstract

Pretest probability (PTP) for assessing obstructive coronary artery disease (ObCAD) was updated to reduce overestimation. However, standard laboratory findings and electrocardiogram (ECG) raw data as first-line tests have not been evaluated for integration into the PTP estimation. Therefore, this study developed an ensemble model by adopting machine learning (ML) and deep learning (DL) algorithms with clinical, laboratory, and ECG data for the assessment of ObCAD. Data were extracted from the electronic medical records of patients with suspected ObCAD who underwent coronary angiography. With the ML algorithm, 27 clinical and laboratory data were included to identify ObCAD, whereas ECG waveform data were utilized with the DL algorithm. The ensemble method combined the clinical-laboratory and ECG models. We included 7907 patients between 2008 and 2020. The clinical and laboratory model showed an area under the curve (AUC) of 0.747; the ECG model had an AUC of 0.685. The ensemble model demonstrated the highest AUC of 0.767. The sensitivity, specificity, and F1 score of the ensemble model ObCAD were 0.761, 0.625, and 0.696, respectively. It demonstrated good performance and superior prediction over traditional PTP models. This may facilitate personalized decisions for ObCAD assessment and reduce PTP overestimation.

## Introduction

Overestimation of patients with obstructive coronary artery disease (ObCAD) should be improved^1,2^. Less than 10% of patients undergoing diagnostic evaluation have ObCAD; the current testing patterns result in up to 50–60% normal coronary angiography (CAG) according to the 2021 American Heart Association (AHA) guidelines^1^. Contemporary pretest probability (PTP), which is based on sex, age, and nature of symptoms, still has a low specificity for identifying patients with ObCAD. The Diamond and Forrester (DF) PTP was updated to define low-risk patients not requiring additional diagnostic testing^2,3^. After PTP estimation, the clinical likelihood can be estimated with the following first-line tests and history of the underlying disease as risk factors for ObCAD: standard laboratory biochemical tests, resting electrocardiogram (ECG), chest radiography, resting echocardiography in selected patients, and if available, coronary artery computed tomography (CT) scan. Those with intermediate likelihood undergo anatomic or functional non-invasive tests based on clinical likelihood, patient characteristics, preference, availability, and local expertise^2^. Posttest probability is estimated with test results and clinical likelihood. Therefore, the optimal estimation of clinical likelihood could act as a gatekeeper to defer a series of diagnostic tests in patients with stable chest pain when the diagnostic yield is low.

Clinical models incorporating ObCAD risk factors, resting ECG changes, and if available, the coronary calcification score can improve the identification of patients with ObCAD compared to PTP^2,4–7^. According to the 2019 European Society of Cardiology guidelines, risk factors for ObCAD such as diabetes, hypertension, dyslipidemia, and smoking could be modifiers of PTP^2^. In particular, the coronary calcium score from coronary artery CT scans was incorporated into the PTP estimate in the 2021 AHA guidelines, despite the incapacity of non-calcified atherosclerotic lesions and weak predictors of ObCAD^1^. However, risk factors, standard laboratory biochemical tests, and resting ECG raw data, which are first-line tests have not been evaluated in terms of their contribution to clinical likelihood; therefore, clinical models incorporating these factors have not been established, although they should be^4^. This may be due to difficulties in the quantification of ECG waveforms and the modeling of correlated data with different characteristics.

This study aimed to develop a clinical model with risk factors and laboratory and ECG waveform data using machine learning (ML) and deep learning (DL) algorithms. Moreover, the performance of this clinical model was evaluated for the assessment of patients with suspected ObCAD.

## Methods

### Data sources and study population

This study was a retrospective observational study of consecutive patients who underwent CAG for suspected ObCAD at the Inha University Hospital, which is a tertiary and university teaching hospital with Regional Cardiocerebrovascular Centers (RCCVCs), established by the Ministry of Health and Welfare in the Incheon district in South Korea. It was approved by the Institutional Review Board of Inha University Hospital (No: 2022-03-012; 14-Mar-2022). The need for informed consent was waived due to the retrospective nature of the study and the use of deidentified data. All procedures were conducted in accordance with the ethical standards of the institutional and/or national research committee and with the 1964 Helsinki declaration and its later amendments or comparable ethical standards.

Patients were eligible if they underwent CAG for suspected ObCAD between October 27, 2008, and August 21, 2020. Those < 18 years, diagnosed with acute myocardial infarction (AMI), or who underwent coronary artery bypass grafting were excluded. AMI was defined using diagnostic codes, procedure codes for insurance claim, and CAG report.

### Data generation

CAG reports and clinical data were extracted from electronic medical records (EMR) and ECG waveform data were extracted from the MUSE data management system (GE Healthcare, USA). The participants were classified into ObCAD and non-ObCAD groups based on CAG results. ObCAD was defined as stenosis with ≥ 50% luminal narrowing of any major vessel on CAG, and non-ObCAD as < 50% narrowing. ObCAD was defined to identify patients whose CAG showed clinically significant stenosis of ≥ 50% and those who could have benefited from further diagnostic tests^5,8,9^.

After extracting the clinical data, the investigators reviewed the literature and reduced dimensionality by selecting relevant features and three derived ratios as risk factors^4,5,8–16^. After eliminating features with > 30% missing values, the final dataset consisted of 27 features: age; sex; body mass index (BMI); systolic blood pressure (SBP); diastolic blood pressure (DBP); history of hypertension, diabetes mellitus, dyslipidemia, and smoking; laboratory findings [white blood cell count, platelet count, hemoglobin, total cholesterol, triglyceride, HDL-cholesterol, LDL-cholesterol, eGFR by CKD-EPI, blood urea nitrogen (BUN), creatinine, glucose, hemoglobin A1c (HbA1c), aspartate transaminase (AST), alanine transaminase (ALT), and high sensitivity C-reactive protein (hs-CRP) levels]; and three ratios (ratio 1, 2, and 3)^4^.

$$\mathrm{Ratio\,}1 = \frac{\mathrm{Monocyte}}{\mathrm{HDL}-\mathrm{cholesterol}}$$

$$\mathrm{Ratio\,}2 = \frac{\mathrm{Lymphocyte}}{\mathrm{Monocyte}}$$

$$\mathrm{Ratio\,}3 =\mathrm{ log}(\frac{\mathrm{Triglyceride}}{\mathrm{HDL}-\mathrm{cholesterol}})$$

Supplementary Table 1 lists the selected and excluded features of the current study with data structure. Some clinical features were excluded owing to the high missing proportion of data among the clinical features suggested to be related to the prediction of or mortality from ObCAD^4,5,8–16^. Additionally, the ECG data used in this study were not ECG features interpreted by cardiologist, but ECG waveform (signal) data.

The digital, standard 10-s, 12-lead ECG data was acquired in the supine position at a sampling rate of 500 Hz using a GE-Marquette machine (Marquette, WI, USA). The ECG was selected in a window of interest; the index date and time were defined as the date and time when CAG started, and the window of interest was defined as the 7 days before the index date. If the patients had multiple ECG waveforms in the window, the most recent ECG waveform was selected. Patients were excluded if they did not have an ECG waveform within the window of interest.

For the comparison of data distribution between the ObCAD and non-ObCAD groups, 31 ECG patterns and 8 quantitative ECG measurements in the machine-provided interpretation were extracted and summarized for each group^17^. The eight ECG measurements were QRS duration, QT, QTc, PR interval, ventricular rate, and the P-, Q-, and T-wave axes. The ECG patterns were classified using the structured statements of machine-provided ECG interpretation based on the standard key phrases in the MUSE data management system. The 8 ECG measurements and 31 patterns are listed in Supplementary Table 2. The ECG patterns and measurement, which were provided by GE machine, were used for the group comparison, but they were not included in the model.

In this study, we proposed a DL model adopting 1D ResNet for ECG raw data, and an ensemble method to combine the ECG DL model and a clinical model. In order to facilitate comparison with our proposed model, a predictive model using ECG measurements and GE-provided interpretations was constructed and compared with the proposed ECG DL model. Furthermore, the proposed ensemble model, combining ECG DL model and clinical models was compared with the ensemble model comprising the clinical model and the predictive model using ECG measurements and GE-provided interpretations.

### Model framework and computing environment

**Figure 1:**
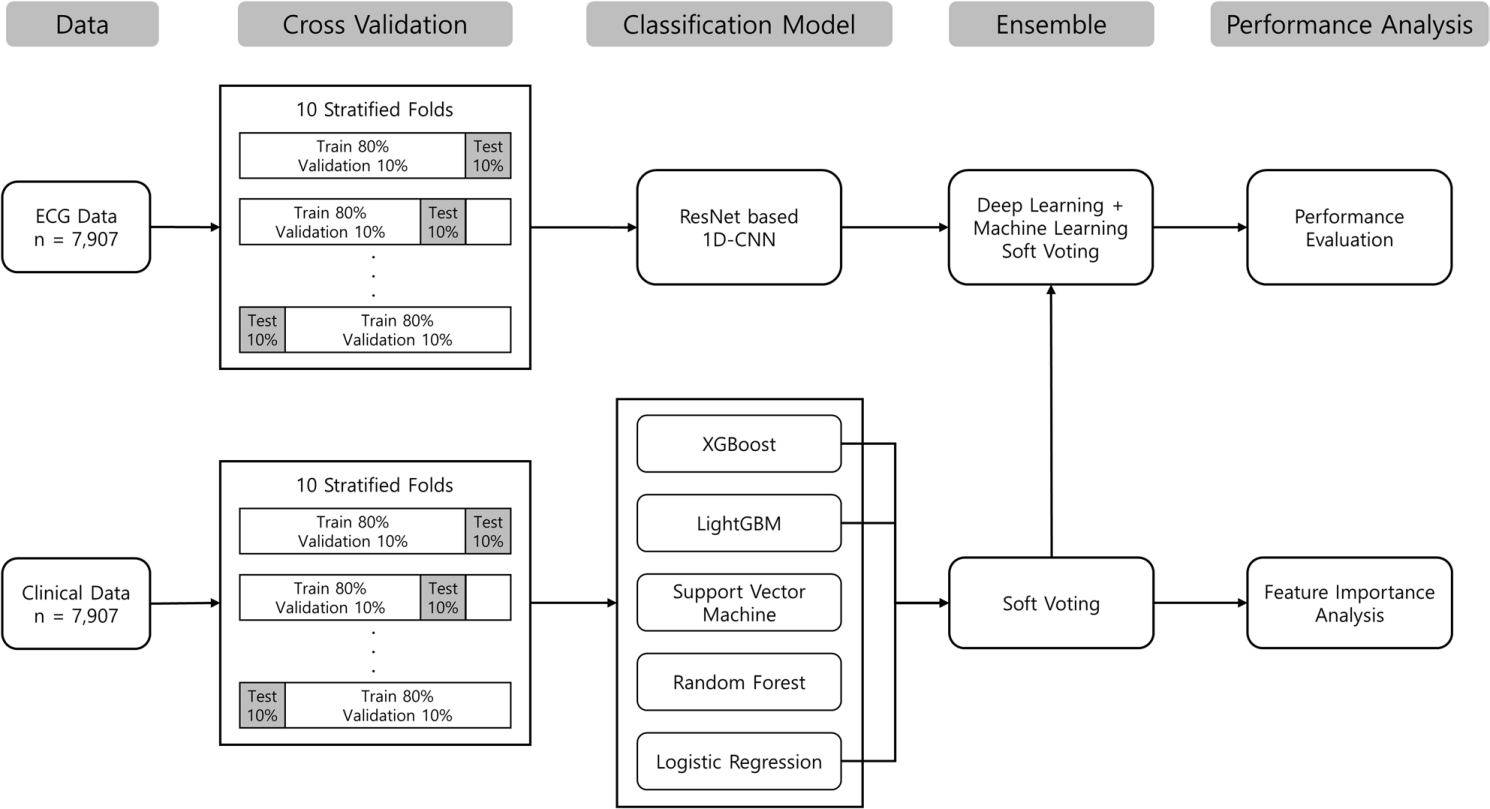
Model framework.

The framework of this study is illustrated in Fig. 1. The developed clinical ML model is an ensemble of LR, LightGBM, and XGBoost using soft-voting, which yields the highest predictive performance for clinical data. Subsequently, the final ensemble model was constructed using soft-voting of the clinical ML and ECG DL models on a ResNet-based 1D-CNN network, which produced the highest performance. The details of each model were provided in the clinical, the ECG and ensemble model development. All the experiments were performed on a computer with GeForce RTX 3090 Ti, i9-12900 K CPU, 64 GB of memory, Python 3.7, and Tensorflow 2.7.

### Clinical model development

The clinical data distribution was evaluated and following pre-processing techniques were applied: correction and removal of inconsistent values, missing value handling, feature encoding, and standardization. After outliers were defined using data distribution and clinical information, they were replaced with missing values for each feature in the clinical dataset. Normalization using Gaussian Rank was applied for logistic regression (LR), support vector machine (SVM), LightGBM, and DL. Additionally, a sensitivity analysis was performed using imputation techniques and the log transformation of the features.

After preprocessing the dataset, five ML algorithms were applied to predict ObCAD based on the clinical features: LR, random forest (RF), SVM, LightGBM, and XGBoost. These five algorithms were selected for the clinical model because they include techniques to suppress overfitting and they are suitable for structured large-scale data and capable of improving predictive power. Specifically, XGBoost, LightGBM, and RF are commonly used models that could prevent overfitting and increase the generalization of the model by adjusting maximum depths and learning rates, and they were known to have high predictive power^18–20^. SVM is the ML algorithm that could solve nonlinear problems using various kernel techniques and maximize the margin to prevent overfitting^21^. Additionally, logistic regression is a linear model that allows for easy interpretation of model coefficients and has low computational complexity, making it suitable for large datasets^22^. To this end, we performed 20 iterations of randomized searches on the key hyperparameters of each clinical model. Among the 20 randomly selected hyperparameters, we fine-tuned the key hyperparameters for each clinical model to the value that achieved the highest AUC in the validation set.

The optimal combination of clinical classifiers that maximized the F1 score was chosen as the selected ensemble output for the clinical ML classifiers (LR, XGBoost, and LightGBM). Initially, we compared the performance of LR, RF, SVM, LightGBM, and XGBoost models, selecting LightGBM with the highest F1 score. Subsequently, the remaining four algorithms were added to the LightGBM model to create two-component ensemble models for comparison. Among these, the ensemble model consisting of LR with LightGBM was chosen. We then repeated the process by adding the remaining three algorithms to the two-component ensemble model, resulting in a three-component ensemble model. However, we terminated the forward selection method when adding another algorithm to the three-component model, as all four-component ensemble models showed inferior performance compared to the three-component model (Supplementary Fig. 1). For the ensemble model of three clinical classifiers, the soft voting method was used to enhance prediction performance. To this end, the soft voting method utilizes the average probability of the classifier output.

The SHAP (SHapley Additive exPlanation) values of the clinical features were calculated in the ensemble model to estimate feature importance^23^. The value is used to determine feature importance by calculating how much each feature contributes to predicting the target value. SHAP value can be calculated for each sample and is computed by creating combinations of multiple features and determining the average change in the outcome based on the presence or absence of a specific feature. To calculate feature importance, the absolute SHAP values for each feature are averaged across all samples. Furthermore, permutation feature importance was also applied the clinical ensemble model.

### ECG and ensemble model development

A residual neural network (ResNet)-based one-dimensional convolutional neural network (1D-CNN) was constructed to model the ECG waveforms after preprocessing the ECG signal data. For the ECG model, the best hyperparameters achieving the highest F1-score were found by grid search in the validation set. The proposed model based on ResNet was selected because it was superior to four other models adopting machine learning (ML) and DL algorithms [LR, RF, long short term memory (LSTM), and transformer] (Supplementary Table 3).

For the ensemble model of ECG and clinical classifiers, the soft voting method was used to enhance prediction performance. We applied a soft-voting technique in the ensemble model, which combines the predicted probabilities from the clinical ML model and the ECG DL model by taking the average probability to make the final decision for any given sample; the ECG DL output was modeled with the ensemble results from the three clinical ML classifiers, giving equal weight to the DL and the ML classifier combination.

### Model evaluation

The clinical, ECG, and final ensemble models were cross-validated using stratified tenfold to estimate the average predictive performance. The dataset was divided into 10 folds with 90% of training set and 10% of test set. The validation set was randomly selected from the train set and the dataset was divided into train, validation, and test sets with an 8:1:1 ratio. The ML and DL models were built using the train set and the validation set was used to optimize the hyperparameters of the models. The test dataset from the remaining 10% of patients, which was not used in the training or validation, was used to evaluate the performance of the ML and DL algorithms. The performances of the ML and DL model were compared to that of the ensemble model using a DeLong Test by comparing the AUCs under two correlated ROC curves: a widely used non-parametric method in a seminal paper developed by DeLong et al. to compare areas under correlated ROC curves by using the theory on generalized U-statistics to generate an estimated covariance matrix^24^.

The performance measures (AUC, sensitivity, specificity, precision, recall, and F1 score) was estimated with a diagnostic threshold. The threshold was selected by grid search for optimal sensitivity–specificity balance in the validation set. Given the outcome of the classification as true negative (TN), false negative (FN), true positive (TP), and false positive (FP), the performance measures were defined as follows:

$$\mathrm{Sensitivity }= \frac{\mathrm{TP}}{\mathrm{FN }+\mathrm{ TP}}$$

$$\mathrm{Specificity }= \frac{\mathrm{TN}}{\mathrm{TN }+\mathrm{ FP}}$$

$$\mathrm{Precision }= \frac{\mathrm{TP}}{\mathrm{TP }+\mathrm{ FP}}$$

$$\mathrm{Negative\,predictive\,value }= \frac{\mathrm{TN}}{\mathrm{TN }+\mathrm{ FN}}$$

$$\mathrm{F}1\mathrm{ Score }= \frac{2 \times \mathrm{ Precision }\times \mathrm{ Recall}}{\mathrm{Precision }+\mathrm{ Recall}}$$

The performance of the final model was compared with that of other traditional models from previous studies. Three recent models for PTP, the coronary artery disease consortium 1/2 score (CAD1/2) and the pooled cohort equations (PCE), were applied to the current dataset and their performance was compared to that of the ensemble model^3,25,26^. These models were fine-tuned to the dataset using random search and a threshold was chosen using the receiver operating characteristic (ROC) curve. CAD1 and CAD2 were modified to exclude the classification of chest pain (typical, atypical, and other) because text extraction from the EMR was not available. The AUC of the ensemble model was compared with those of the traditional PTP models using a DeLong Test: CAD1, CAD2 and PCE^24^. Additionally, we reviewed the medical records and classified chest pain into typical, atypical, and other in order to compare the final model with original CAD1 and CAD2. The performance of the ensemble model was also compared with those of the original CAD1 and CAD2.

Univariate descriptive statistics were used to compare the characteristics of the ObCAD and non-ObCAD groups. The chi-square test was used to compare the characteristics of categorical variables between the groups. Student’s t- and Mann–Whitney U- tests were used to compare the characteristics of parametric and nonparametric continuous variables between the two groups, respectively.

The proposed framework was implemented using Keras (version 2.0; François Chollet) and TensorFlow (Google; Mountain View, CA, USA). LR, RF, and SVM were provided by the scikit-learn library. In the descriptive statistics, a two-tailed *p-value* of < 0.05 was considered statistically significant.

### Ethics statement

This study was approved by the Institutional Review Board of Inha University Hospital (No: 2022–03-012; 14-Mar-2022). The need for informed consent was waived due to the retrospective nature of the study and the use of deidentified data by the Institutional Review Board of Inha University Hospital.

## Results

**Figure 2:**
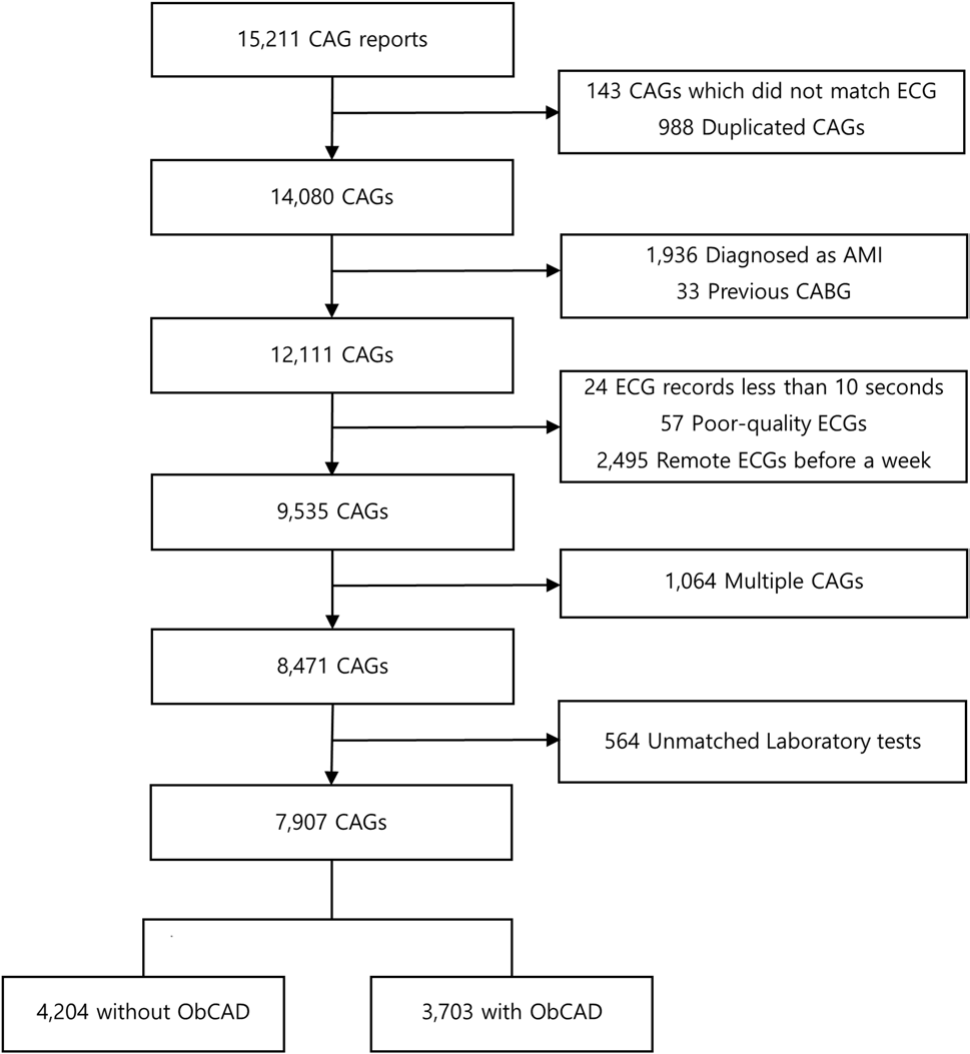
Flowchart of the data used in the study. *CAG* coronary angiography, *ECG* electrocardiogram, *AMI* acute myocardial infarction, *CABG* coronary artery bypass graft surgery, *ObCAD* obstructive coronary artery disease.

Among 15,211 CAGs, 988 were eliminated as they were duplicates, and 14,080 were included with matched ECG waveforms were utilized with the DL algorithm. data (Fig. 2). Those who were diagnosed with AMI or underwent coronary artery bypass graft surgery were excluded. After excluding those with poor-quality ECG waveforms , those without ECG waveforms in the time window, or ECG waveforms recorded for ≥ 10 s, 9,535 CAGs remained. A single recent CAG per patient was selected if the patient underwent multiple CAGs during the study period, and 564 CAGs were excluded because no biochemical laboratory testing was conducted during the window of interest. Finally, 7,907 CAGs were retained for the analysis.

Those with stenosis ≥ 50% were more likely to be older than those with stenosis < 50% (Table 1). The proportion of men was higher in the ObCAD group than in the non-ObCAD group. The ObCAD group had a higher prevalence of diabetes mellitus or hypertension than the non-obCAD group, and were more likely to be current or past smokers. Laboratory findings with significant differences between the ObCAD and non-ObCAD groups were selected, that included all except AST and ALT.

**Table 1:** Characteristics of the study population and clinical data distribution.

	Total (N = 7907)	Non-ObCAD (N = 4204)	ObCAD (N = 3703)	*p*-value
Demographic characteristics				
Age, years	63.6 (12.5)	60.9 (12.9)	66.7 (11.4)	< 0.01
Male	4847 (61.3)	2304 (54.8)	2544 (68.7)	< 0.01
Medical history and social history				
Diabetes mellitus	2420 (30.6)	933 (22.2)	1485 (40.1)	< 0.01
Hypertension	4515 (57.1)	2144 (51.0)	2370 (64.0)	< 0.01
Dyslipidemia	1083 (13.7)	584 (13.9)	500 (13.5)	0.66
Smoking				< 0.01
Nonsmoker	5005 (63.3)	2804 (66.7)	2207 (59.6)	
Past smoker	981 (12.4)	446 (10.6)	533 (14.4)	
Current smoker	1921 (24.3)	954 (22.7)	967 (26.1)	
Physical measurements				
Systolic blood pressure, mmHg	137.7 (21.6)	136.5 (21.4)	138.9 (21.8)	0.03
Diastolic blood pressure, mmHg	81.4 (13.5)	82.0 (13.5)	80.7 (13.5)	< 0.01
Body mass index, kg/m^2^	24.8 (3.6)	24.9 (3.7)	24.6 (3.6)	< 0.01
Laboratory findings				
White blood cell count, × 10^9^/L	6.5 (5.3–8.1)	6.3 (5.2–7.8)	6.8 (5.6–8.5)	< 0.01
Hemoglobin, g/dL	13.5 (2.0)	13.7 (1.9)	13.3 (2.1)	< 0.01
Platelet count, × 10^9^/L	227.7 (65.3)	230.6 (63.3)	224.1(67.4)	< 0.01
Total cholesterol, mg/dL	163 (136–192)	170 (143–196)	156 (128–187)	< 0.01
Triglyceride, mg/dL	115 (83–166)	112 (80–164)	120 (86–169)	< 0.01
LDL cholesterol, mg/dL	99 (75–127)	104 (81–130)	93 (70–122)	< 0.01
HDL cholesterol, mg/dL	45 (38–55)	48 (39–58)	43 (36–41)	< 0.01
BUN, mg/dL	15.4 (12.3–19.7)	14.8 (12.0–18.6)	16.1 (12.8–21.2)	< 0.01
Creatinine, mg/dL	0.9 (0.8–1.1)	0.9 (0.7–1.0)	1.0 (0.8–1.2)	< 0.01
eGFR by CKD-EPI, mL/min/1.73 m^2^	86 (69–97)	90 (74–99)	82 (61–93)	< 0.01
Glucose, mg/dL	110 (97–138)	107 (96–129)	116 (100–150)	< 0.01
HbA1c, %	5.9 (5.6–6.6)	5.8 (5.5–6.3)	6.1 (5.7–7.0)	< 0.01
AST, IU/L	23 (19–31)	23 (19–31)	23 (19–31)	0.33
ALT, IU/L	21 (15–31)	21 (16–31)	21 (15–31)	0.13
Ratio 1	0.8 (0.5–1.1)	0.8 (0.5–1.0)	1.0 (0.7–1.2)	< 0.01
Ratio 2	4.9 (3.5–6.4)	5.2 (3.8–6.8)	4.5 (3.3–6.0)	< 0.01
Ratio 3	0.9 (0.5–1.4)	0.9 (0.4–1.3)	1.0 (0.6–1.5)	< 0.01
hsCRP, mg/dL	0.1 (0.1–0.6)	0.1 (0.1–0.5)	0.2 (0.1–0.7)	< 0.01

Values are presented as means (standard deviations) or medians (interquartile ranges) for continuous variables and proportions (%) for categorical variables.

*LDL* low density lipoprotein, *HDL* high density lipoprotein, *eGFR* estimated glomerular filtration rate, *CKD-EPI* chronic kidney disease epidemiology collaboration, *HbA1c* hemoglobin A1c, *AST* aspartate aminotransferase, *ALT* alanine transaminase, *hsCRP* high sensitivity C-reactive protein.

Ratio 1: monocyte/HDL cholesterol, ratio 2: lymphocyte/monocyte, ratio 3: log(triglyceride/HDL-cholesterol).

The ECG measurements and interpretations provided by the standard 12-lead ECG machine for each group are listed in Table 2. The QRS duration, QT, QTc interval, and PR interval were longer in the ObCAD group than in the non-ObCAD group. The ECG pattern was ‘normal’ in 31.1% of the non-ObCAD group and 20.7% of the ObCAD group. Findings suggestive of ischemia were seen in 14.9% of the patients with ObCAD and only in 11.1% of the patients in the non-ObCAD group. Moreover, using the traditional interpretation, the prevalences of right bundle branch block, left ventricular hypertrophy, first-degree atrioventricular block, and findings suggestive of prior infarction were significantly different between those with and without ObCAD.

**Table 2:** Characteristics of electrocardiogram data distribution.

	Total (N = 7907)	Non-ObCAD (N = 4204)	ObCAD (N = 3703)	*p*-value
Measurement of electrocardiographic features				
QRS duration, ms	92 (86–102)	92 (86–102)	94 (86–104)	< 0.01
QT, ms	400 (376–426)	400 (376–424)	402 (378–428)	< 0.01
QTc, ms	435 (415–460)	434 (414–458)	437 (416–462)	< 0.01
PR interval, ms	164 (150–182)	164 (148–180)	166 (150–182)	< 0.01
Ventricular rate, bpm	71 (62–82)	71 (63–82)	41 (62–82)	0.50
P axis, ˚	52 (35–63)	52 (35–64)	52 (36–63)	0.55
R axis, ˚	31 (4–58)	35 (8–60)	27 (-1–55)	< 0.01
T axis, ˚	47 (25–69)	44 (24–63)	51 (26–78)	< 0.01
Machine-provided interpretation				
Normal	26.3%	31.1%	20.7%	< 0.01
Left bundle branch block	1.7%	1.9%	1.4%	0.08
Incomplete left bundle branch block	0.3%	0.33%	0.24%	0.46
Right bundle branch block	4.6%	4.1%	5.2%	0.02
Incomplete right bundle branch block	1.6%	1.2%	2.0%	0.01
Complete heart block	0.1%	0.17%	0.08%	0.35
Atrial fibrillation	6.1%	6.9%	5.2%	< 0.01
Atrial flutter	0.4%	0.5%	0.4%	0.48
Acute myocardial infarction	3.2%	2.3%	4.2%	< 0.01
Left ventricular hypertrophy	10.2%	9.0%	11.5%	< 0.01
Premature ventricular contractions	3.7%	3.7%	3.8%	0.69
Premature atrial contractions	2.4%	2.3%	2.4%	0.77
First-degree atrioventricular block	5.3%	4.6%	6.2%	< 0.01
Second-degree atrioventricular block	0.1%	0.12%	0.08%	0.73
Fascicular block	1.6%	1.6%	1.6%	0.86
Sinus bradycardia	16.3%	15.4%	17.3%	0.02
Other bradycardia	0.2%	0.17%	0.22%	0.61
Sinus tachycardia	5.1%	4.9%	5.3%	0.49
Ventricular tachycardia	0.04%	0.00%	0.08%	0.10
Supraventricular tachycardia	0.05%	0.02%	0.08%	0.35
Prolonged QT	7.4%	7.6%	7.2%	0.42
Pacemaker	0.1%	0.10%	0.08%	0.99
Ischemia	12.9%	11.1%	14.9%	< 0.01
Low QRS voltage	1.8%	1.5%	2.1%	0.06
Intraventricular block	0.7%	0.6%	0.8%	0.20
Prior infarct	14.4%	8.8%	20.7%	< 0.01
Nonspecific T-wave abnormality	8.1%	8.3%	7.8%	0.40
Nonspecific ST abnormality	4.3%	3.6%	5.0%	< 0.01
Left axis deviation	6.0%	5.3%	6.9%	< 0.01
Right axis deviation	0.3%	0.3%	0.2%	0.13
Early repolarization	1.5%	2.0%	1.0%	< 0.01

Values are presented as median (interquartile range) for continuous variables and proportion (%) for categorical variables.

**Figure 3:**
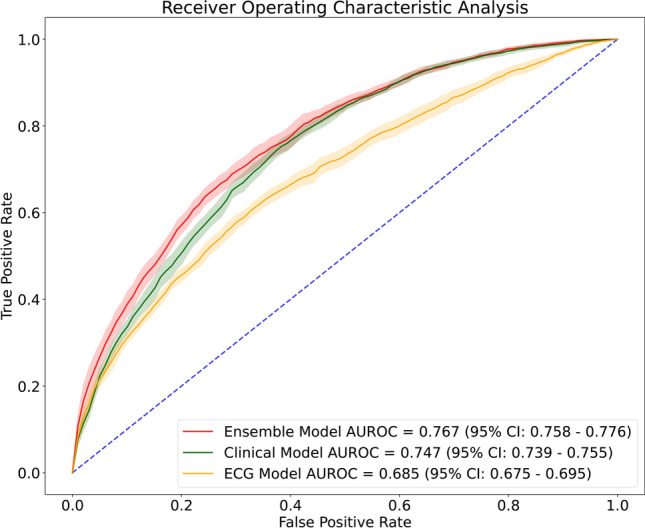
ROC curves for the electrocardiogram, clinical and ensemble prediction models using the test dataset. *ECG* electrocardiogram, *ROC* receiver operating characteristic.

Table 3 lists the performance of the ECG, clinical, and ensemble models for the assessment of ObCAD. The AUC of the ensemble clinical model was significantly higher than that of the ECG and clinical models (*p*-value < 0.05 for both comparisons). The AUC, sensitivity, specificity, precision, and negative predictive value of the ensemble model for ObCAD screening were 0.767, 0.761, 0.625, 0.642 and 0.749, respectively, in the test set. The F1 score in the ensemble model was 0.696, which was higher than the 0.617 and 0.691 scores in the ECG and clinical models, respectively. Figure 3 presents the ROC curves of the ECG, clinical, and ensemble models.

**Table 3:** Performance of the clinical, ECG, and ensemble prediction models.

	ECG model	Clinical model	Ensemble model
AUROC	0.685 (0.675–0.695)	0.747 (0.739–0.755)	0.767 (0.758–0.776)
Sensitivity	0.636 (0.586–0.686)	0.774 (0.761–0.787)	0.761 (0.738–0.784)
Specificity	0.629 (0.573–0.686)	0.591 (0.578–0.604)	0.625 (0.600–0.651)
Precision	0.607 (0.584–0.630)	0.624 (0.617–0.633)	0.642 (0.628–0.657)
Negative predictive value	0.665 (0.653–0.677)	0.748 (0.737–0.759)	0.749 (0.734–0.765)
F1 Score	0.617 (0.599–0.634)	0.691 (0.683–0.699)	0.696 (0.683–0.709)

*AUROC* area under the receiver operating characteristic curve.

**Figure 4:**
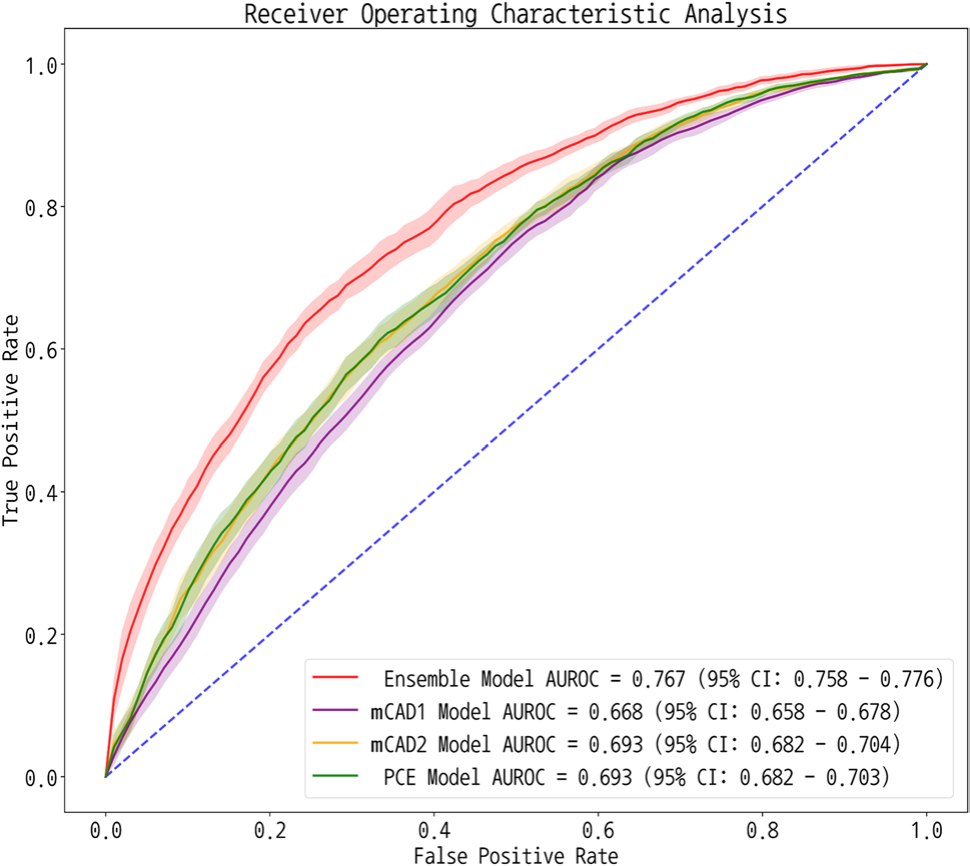
ROC curves for the ensemble prediction model with modified traditional models. *ROC* receiver operating characteristic, *CAD1* coronary artery disease consortium 1 score, *CAD2* coronary artery disease consortium 2 score, *PCE* pooled cohort equation.

Figure 4 and Supplementary Table 4 show the comparison of the ROC curve of the ensemble model with those of the modified CAD1, CAD2, and PCE models after using the datasets in them. The AUC of the ensemble model was 0.767, which was higher than that of modified CAD1, CAD2, and PCE (0.668, 0.693, and 0.693, respectively); each comparison was statistically significant with *p*-value < 0.05. Similarly, the ensemble model outperformed both the original CAD1 and CAD2 significantly after reviewing and classifying chest pain (Supplementary Table 5). The original CAD1 achieved an AUC of 0.633 and an F1 score of 0.601, while the original CAD2 achieved an AUC of 0.693 and an F1 score of 0.630. The ensemble model showed significantly higher AUC than the original CAD1 and CAD2 (*p*-value < 0.05 for both comparisons).

Our proposed models were compared with corresponding predictive models using ECG measurements and GE-provided interpretations (Supplementary Table 6). The proposed ECG model with 1D ResNet was superior to the predictive model utilizing ECG measurements and GE-provided interpretations. The ML model using LR achieved an F1 score of 0.566 and an AUC of 0.621, while the DL model achieved an F1 score of 0.617 and an AUC of 0.685. Additionally, the proposed ensemble model, combining ECG DL clinical and clinical models, outperformed the corresponding ensemble model comprising the clinical model and the predictive model using ECG measurements and GE-provided interpretations (*p*-value < 0.05).

**Figure 5:**
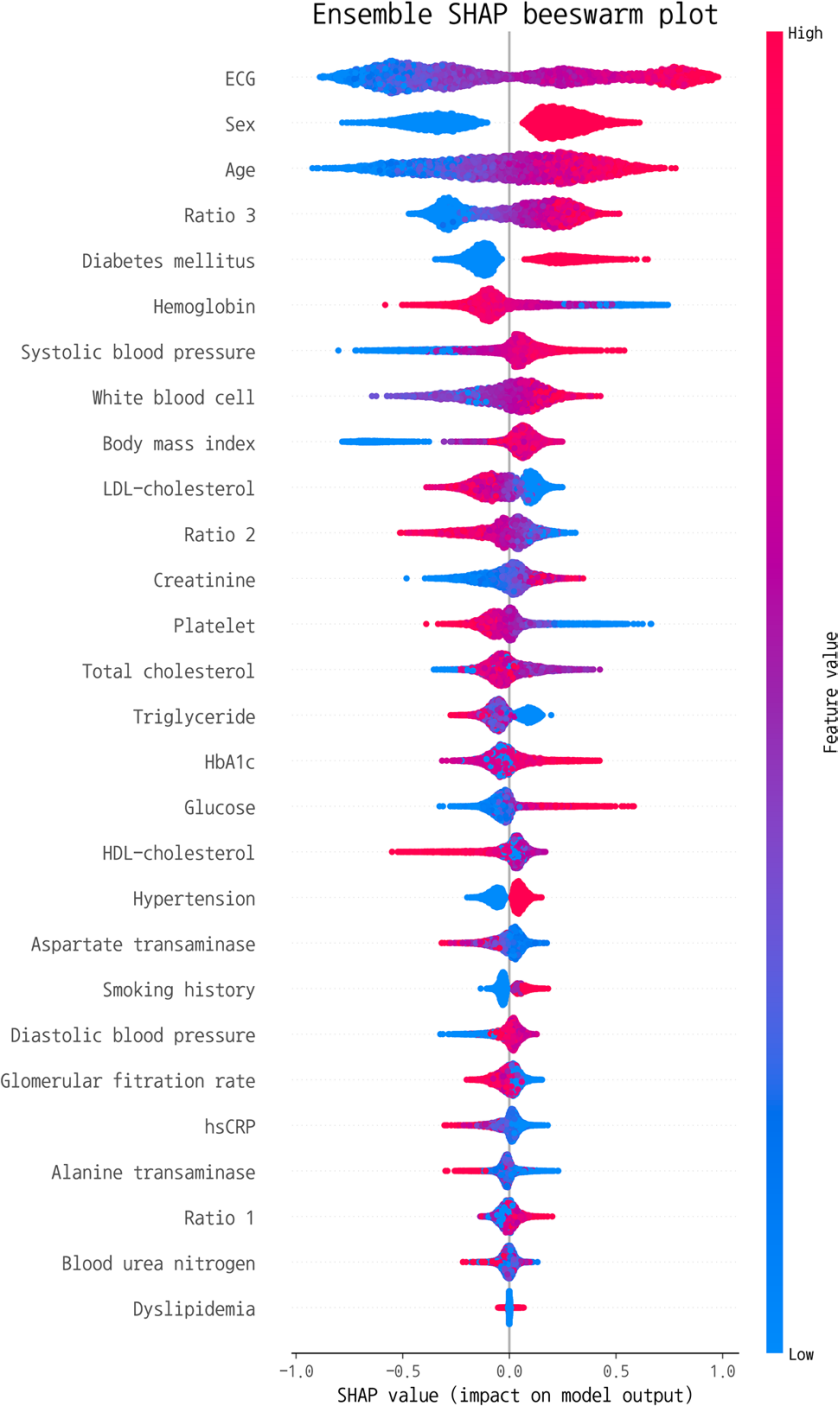
SHAP plot for ECG and the clinical features. *LDL* low density lipoprotein, *HDL* high density lipoprotein, *HbA1c* hemoglobin A1c, *hsCRP* high sensitivity C-reactive protein. Ratio 1: monocyte/HDL cholesterol, ratio 2: lymphocyte/monocyte, ratio 3: log(triglyceride/HDL-cholesterol).

The SHAP values of the ensemble model are shown in Fig. 5. To visualize the importance of features in an ensemble model composed of heterogeneous data, such as clinical data and ECG signals, we measured the SHAP values using the clinical data and the ObCAD probability of the ECG DL model. In the ensemble model, ECG emerged as the most influential variable, followed by the top 10 clinical characteristics with the greatest contribution to the prediction model: sex, age, ratio 3, diabetes, hemoglobin, SBP, white blood cell count, BMI, LDL-cholesterol, and ratio 2. The analysis of SHAP values revealed that male sex, advanced age, high ratio 3, diabetes, low hemoglobin levels, elevated SBP, leukocytosis, obesity, and low ratio 2 were associated with ObCAD. Furthermore, Supplementary Fig. 2 presents the permutation feature importance of the clinical ensemble model. The top 10 clinical features were found to be age, sex, ratio 3, BMI, triglyceride, hemoglobin, white blood cell count, diabetes, LDL-cholesterol, and total cholesterol. Notably, age, sex, and ratio 3 consistently emerged as the top three features in both the SHAP and permutation feature importance analyses. Furthermore, the other common features among the two methods were BMI, hemoglobin, white blood cell count, diabetes, and LDL-cholesterol. However, the permutation method identified additional features, triglyceride and total cholesterol as influential features, while the SHAP analysis highlighted ratio 2 and SBP.

## Discussion

We developed a DL- and ML-based ensemble model that includes clinical, laboratory, and ECG waveform data for the assessment of ObCAD. This model utilized the information from the first-line tests (clinical and ECG data) with DL and ML algorithms, and enhanced the performance by combining this information in an ensemble model. The ensemble model was superior to the traditional risk estimations and could be integrated into clinical practice. Additionally, it may contribute to reducing unnecessary anatomic or functional diagnostic tests for the assessment of ObCAD if the performance of the ensemble model should be innovatively enhanced in the future.

This study aimed to build the predictive model using the first-line tests (basic clinical, laboratory and ECG data) to improve the estimation of clinical likelihood before anatomic and functional tests, and tried to enhance objectivity using ECG waveforms. Previous research used treadmill test and myocardial Single Photon Emission Computed Tomography (SPECT) as predictors in the models^27,28^. Therefore, those models could not be applied with first-line tests, and the functional tests are less fast and less affordable. Moreover, raw ECG data as the first-line tests, have been little evaluated in predictive models that employ DL algorithms for assessing ObCAD. Most of previous studies utilized the ECG features from human interpretation in a few open databases according to a recent review study: the Heart Disease dataset in UC Irvine Machine Learning Repository^29^, the St. Petersburg INCART 12-lead Arrhythmia Database in PhysioNet^30^, and the Fantasia database in PhysioNet^29^. In the review study, out of eight research studies, only six studies utilized ECG waveforms with ML algorithms for CAD detection, with a particular focus on longer ECG signals like 15-min ECG or 24-h Holter monitoring, as opposed to the standard 12-lead ECG used in routine practice^31–34^. Therefore, previous studies could not be adopted into clinical practice and the ECG data themselves could not be utilized without human interpretation.

Previous studies have suggested that laboratory-based models are superior to PTP^4,5^. In particular, a recent study demonstrated that a laboratory-based model of eight variables, including age, sex, type of chest pain, hypertension, type 2 diabetes mellitus, smoking, LDL-C, and creatinine, showed a higher AUC than the DF, CAD1/2, and Duke clinical scores did^5^. Furthermore, a predictive model consisting of laboratory, clinical, and ECG characteristics with good performance was developed for predicting the SYNTAX score of CAG result^4^; family history of ObCAD, past history of peripheral vascular disease, ST-T changes in ECG and selected standard laboratory tests were included. The model’s F1 score was 0.71 and could differentiate between zero and non-zero SYNTAX scores.

Diverse sources of information and a plethora of methodologies can enhance personalized decisions for risk assessment of ObCAD^4^. A recent study reviewed the literature on the risk stratification score for predicting long-term cumulative mortality of ObCAD and suggested that the future of mortality prediction should be developed by combining clinical risk predictors and cardiovascular imaging, which has the highest predictive accuracy^10^. Similarly, combining four different risk scores showed a better reclassification index relative to each score: The Global Registry for Acute Coronary Events; SYNTAX; residual SYNTAX; and the age, creatinine, and ejection fraction score^13^. To enhance predictive performance, data-driven analytical solutions, such as ML or DL algorithms, have been increasingly applied to diverse datasets with computational power. In the current study, the ensemble ML and DL classifiers, including clinical, laboratory, and ECG data, were superior to traditional risk estimation models. In this study, our model achieved higher performance by utilizing physical measurement, ECG data and more clinical variables compared to the traditional PTP (CAD1, CAD2, and PCE) that only included sex, age, past medical history in common, and total-cholesterol, and HDL-cholesterol additionally. Moreover, to improve the performance of our proposed model, we used the ensemble method from various algorithms in addition to the LR used in the comparison models; the ensemble method was designed to leverage diversity in data or algorithms to improve performance. Therefore, this model may achieve higher performance relative to the conventional PTP by using various data and algorithms in the clinical model and the ECG model.

Although the ensemble model was superior to the traditional risk stratification, the ECG, clinical and ensemble model showed modest performances to assess ObCAD and it require further improvement for practical use. In our previous study, the ECG model demonstrated fair performance in suggesting the probability of ObCAD, whereas it showed excellent performance in detecting AMI^35^. It may be because ObCAD is the progressive narrowing of coronary arteries with no ECG characteristics or subtle, while AMI is related to myocardial necrosis and more obvious ECG change due to acute obstruction of coronary artery. Similarly, AMI results in high level of cardiac enzyme at admission or increase by time, which could make the clinical model have higher discrimination. Other researchers demonstrated that the ECG model showed completely different AUC between two subgroups of diagnosis: 0.973 in AMI and 0.566 in ObCAD^36^. Therefore, the ECG and clinical models for assessing ObCAD have to deal with more complex and daunting task compared to those for diagnosing AMI; if these models are enhanced in sophisticated and innovative ways, the ensemble model may help clinicians make better discrimination and decision for initial assessment of ObCAD.

According to the SHAP values, male sex and advanced age were the most predictive clinical features in the ML model. Sex and age, which is the determinant of ObCAD prevalence, is also the component of the DF PTP and all other PTP models: CAD1, CAD2, and PCE models. They were followed by ratio 3 called the atherogenic index of plasma (AIP). In the other ML model for the estimation of PTP, hypercholesterolemia and HDL-cholesterol followed age and sex^37^. In contrast, our findings showed that AIP contributed more than each component of the ratio (triglyceride and HDL-cholesterol) and history of dyslipidemia. However, direct comparison was not available because they included only total, HDL-cholesterol and LDL-cholesterol as predictors in their model. We included three composite markers in the model proposed as novel marker of ObCAD. Ratio 1 consisted of monocyte and HDL-cholesterol ratio which involve inflammation and atherosclerotic plaque formation^38^. Monocyte is known to play fundamental roles in inflammation and the activation of monocyte is an important initial step in the development of ObCAD, while HDL-cholesterol could prevent inflammation by directly acting on monocyte^38^. Thus, monocyte to HDL-cholesterol ratio is suggested as a novel marker to assess the inflammation in atherosclerosis. Thus, previous research suggested that high monocyte to HDL-cholesterol ratio is associated with high SYNTAX score in stable ObCAD, and the CAD severity and cardiovascular mortality in acute coronary syndrome^39,40^. Similarly, low lymphocyte to monocyte ratio (ratio 2) has been found to be a novel systemic inflammatory marker and may provide additive information in the assessment of cardiovascular risk, although it remains inconclusive due to insufficient number of previous studies^41–43^. In this study, the SHAP plot revealed an association between ObCAD and low ratio 2 along with high white blood cell count (WBC), both of which are indicators of inflammation. Lastly, the logarithm of triglyceride to HDL-cholesterol (ratio 3) is associated with the burden of atherosclerosis^44^. High triglyceride and low HDL-cholesterol is known as atherogenic dyslipidemia and is correlated with the metabolic syndrome, insulin resistance, and atherosclerotic cardiovascular disease risk^14^.

This study included a contemporary population with suspected ObCAD and who received CAG. Therefore, there is little risk of misclassification and the study is based on the real world ECG waveform data. Furthermore, the current model could help to reduce unnecessary diagnostic tests in the current practice. However, this study has some limitations. The performance of the ensemble model should be further enhanced for practical use in the future because the performance of the model was modest. Furthermore, it included only those who underwent CAG and not all patients with suspected ObCAD because the patient’s CAG data were extracted from the EMR. Therefore, in the future, the model should be re-evaluated in all patients with suspected ObCAD for validation and generalization. Finally, unstable angina was not excluded along with AMI and the inclusion criteria was angina pectoris because the data were extracted from the EMR. In the EMR, diagnosis of angina pectoris and ObCAD could not differentiate between stable and unstable angina.

## Conclusion

A predictive model with laboratory, clinical, and ECG data was developed and internally validated. It demonstrated good performance which was superior to that of the traditional PTPs. With further enhancement, this predictive model may facilitate the selection of patients who would benefit most from further diagnostic assessment for ObCAD. However, its clinical utility should be further validated externally by use in the clinical field.

### Supplementary Information

Supplementary Information.

## Data Availability

The data that support the findings of this study are available from Inha University Hospital but restrictions apply to the availability of these data, which were used under license for the current study, and so are not publicly available. Data are however available from the authors upon reasonable request and with permission of Inha University Hospital.
