# Supplementary material for: Machine learning approaches that use clinical, laboratory, and electrocardiogram data enhance the prediction of obstructive coronary artery disease
Source: Sci Rep. 2023 Aug 3;13:12635. doi: 10.1038/s41598-023-39911-y (PMC10400607; doi:10.1038/s41598-023-39911-y)
Supplement: Supplementary file 1 — Supplementary Information. [file 41598_2023_39911_MOESM1_ESM.docx]

Supplementary information

**Machine learning approaches that use clinical, laboratory, and electrocardiogram data enhance the prediction of obstructive coronary artery disease**

**Hyun-Gyu Lee^1^, Sang-Don Park^2^, Jang-Whan Bae^3^, SungJoon Moon^4^, Chai Young Jung**^5^**, Mi-Sook Kim^6^, Tae-Hun Kim^7^, Won Kyung Lee^8*^**

^1^School of Medicine, Inha University, Incheon, Korea

^2^Department of Cardiology, Inha University Hospital, School of Medicine, Inha University, Incheon, Korea

^3^Division of Cardiology, Department of Internal Medicine, Chungbuk National University College of Medicine, Cheongju, Korea

^4^ApexAI, Korea

^5^Biomedical Research Institute, Inha University Hospital, Incheon, Korea

^6^Division of Clinical Epidemiology, Medical Research Collaborating Center, Biomedical Research Institution, Seoul National University Hospital, Seoul, Korea

^7^Department of Artificial Intelligence, Inha University, Incheon, Korea

^8^Department of Prevention and Management, Inha University Hospital, School of Medicine, Inha University, Incheon, Korea

*Corresponding author:

**Won Kyung Lee**

27 Inhang-Ro, Jung-Gu, Inha University Hospital, Incheon, Republic of Korea

Telephone: +82-32-890-2885

Mobile: +82-10-6360-7965

Fax: +82-32-890-3459

Supplementary Table 1. Structure of data with selected and excluded features

| Model | Predictors | Selected features | Excluded features |
| --- | --- | --- | --- |
| Clinical model | Demographics | Age  Sex |  |
|  | Risk factors | Diabetes mellitus  Hypertension  Dyslipidemia  Smoking |  |
|  | Physical measurements | Systolic blood pressure  Diastolic blood pressure  Body mass index | Abdominal circumference |
|  | Laboratory tests | White blood cell  Hemoglobin  Platelet  Total cholesterol  Triglyceride  LDL cholesterol  HDL cholesterol  Blood urea nitrogen  Creatinine  eGFR by CKD-EPI  Glucose  HbA1c  AST  ALT  Ratio 1 (monocyte/ HDL-cholesterol)  Ratio 2 (lymphocyte/monocyte)  Ratio 3 [log(triglyceride/HDL-cholesterol)]  High-sensitivity C-reactive protein | High-sensitivity cardiac troponin I  High-sensitivity cardiac troponin T  ProBNP  Adiponectin  Kidney injury molecule-1  Renin  Endothelin-1  Lipoprotein  Metabolites |
| ECG model | ECG data | ECG waveform (signal) data | ECG features  ECG patterns  ECG measurements |

LDL, low density lipoprotein; HDL, high density lipoprotein; eGFR, estimated glomerular filtration rate; CKD-EPI, chronic kidney disease epidemiology collaboration; HbA1c, Hemoglobin A1c; AST, aspartate aminotransferase; ALT, alanine transaminase; ProBNP, pro B-type natriuretic peptide

Supplementary Table 2. ECG features in the machine-provided interpretation

| **ECG features for comparison** | |
| --- | --- |
| **8 Measurements** | QRS duration (ms)  QT (ms)  QTc (ms)  PR interval (ms)  Ventricular rate (bpm)  P axis (˚)  R axis (˚)  T axis (˚) |
| **31 Patterns** | Normal  Left bundle branch block  Incomplete left bundle branch block  Right bundle branch block  Incomplete right bundle branch block  Complete heart block  Atrial fibrillation  Atrial flutter  Acute myocardial infarction  Left ventricular hypertrophy  Premature ventricular contractions  Premature atrial contractions  First-degree atrioventricular block  Second-degree atrioventricular block  Fascicular block  Sinus bradycardia  Other bradycardia  Sinus tachycardia  Ventricular tachycardia  Supraventricular tachycardia  Prolonged QT  Pacemaker  Ischemia  Low QRS voltage  Intraventricular block  Prior infarct  Nonspecific T-wave abnormality  Nonspecific ST abnormality  Left axis deviation  Right axis deviation  Early repolarization |

Supplementary Table 3. Performance of ECG models according to algorithms

|  | AUC | Sensitivity | Specificity | Precision | NPV | F1-score |
| --- | --- | --- | --- | --- | --- | --- |
| Logistic Regression | 0.528 | 0.532 | 0.509 | 0.489 | 0.552 | 0.509 |
| Bi-LSTM | 0.515 | 0.514 | 0.526 | 0.488 | 0.552 | 0.498 |
| Random Forest | 0.526 | 0.382 | 0.670 | 0.505 | 0.552 | 0.435 |
| Transformer | 0.661 | 0.588 | 0.655 | 0.603 | 0.645 | 0.593 |
| 1D ResNet | 0.685 | 0.636 | 0.629 | 0.607 | 0.665 | 0.617 |

LSTM, long short term memory; NPV, Negative Predictive Value

Supplementary Table 4. Performance of modified traditional prediction models

|  | **mCAD1** | **mCAD2** | **PCE** | **Ensemble Model** |
| --- | --- | --- | --- | --- |
| AUROC | 0.668  (0.658 – 0.678) | 0.693  (0.682 – 0.704) | 0.693  (0.682 – 0.703) | 0.767  (0.758 – 0.776) |
| Sensitivity | 0.654  (0.618 – 0.689) | 0.665  (0.630 – 0.701) | 0.665  (0.615 – 0.716) | 0.761  (0.738 – 0.784) |
| Specificity | 0.586  (0.561 – 0.631) | 0.609  (0.574 – 0.643) | 0.608  (0.561 – 0.655) | 0.625  (0.600 – 0.651) |
| Precision | 0.582  (0.576 – 0.589) | 0.601  (0.588 – 0.613) | 0.601  (0.588 – 0.615) | 0.642  (0.628 – 0.657) |
| Negative predictive value | 0.659  (0.645 – 0.674) | 0.675  (0.660 – 0.690) | 0.677  (0.659 – 0.796) | 0.749  (0.734 – 0.765) |
| F1 score | 0.615  (0.598 – 0.631) | 0.630  (0.616 – 0.644) | 0.629  (0.608– 0.649) | 0.696  (0.683 – 0.709) |

mCAD1, modified coronary artery disease consortium 1 score; mCAD2, modified coronary artery disease consortium 2 score; PCE, pooled cohort equation; AUROC, area under the receiver operating characteristic curve

Supplementary Table 5. Performance of traditional prediction models without modification

|  | **CAD1** | **CAD2** |
| --- | --- | --- |
| AUROC | 0.633 (0.621– 0.645) | 0.693 (0.682 – 0.703) |
| Sensitivity | 0.645 (0.622 – 0.668) | 0.663 (0.635 – 0.690) |
| Specificity | 0.560 (0.536 – 0.584) | 0.611 (0.579 – 0.644) |
| Precision | 0.564 (0.554 – 0.573) | 0.602 (0.589 – 0.615) |
| Negative predictive value | 0.642 (0.631 – 0.653) | 0.674 (0.663 – 0.684) |
| F1 score | 0.601 (0.590 – 0.612) | 0.630 (0.619 – 0.640) |

CAD1, coronary artery disease consortium 1 score; CAD2, coronary artery disease consortium 2 score; AUROC, area under the receiver operating characteristic curve

Supplementary Table 6. Comparison of proposed models and prediction models from ECG measurement and machine-provided interpretation

|  | **ECG model** | | | **ECG and Clinical ensemble model** |
| --- | --- | --- | --- | --- |
|  | **Logistic Regression** | **Light GBM** | **XGBoost** |  |
| AUROC | 0.621  (0.608 – 0.634) | 0.629  (0.616 – 0.642) | 0.633  (0.620 – 0.645) | 0.753  (0.743 – 0.762) |
| Sensitivity | 0.572  (0.547 – 0.597) | 0.533  (0.505 – 0.562) | 0.550  (0.517– 0.584) | 0.714  (0.681 – 0.747) |
| Specificity | 0.603  (0.571 – 0.636) | 0.646  (0.624 – 0.668) | 0.635  (0.609 – 0.660) | 0.653  (0.626 – 0.680) |
| Precision | 0.561  (0.546 – 0.576) | 0.570  (0.562 – 0.579) | 0.570  (0.559 – 0.582) | 0.645  (0.635 – 0.656) |
| Negative predictive value | 0.616  (0.605 – 0.626) | 0.612  (0.602 – 0.621) | 0.617  (0.604 – 0.629) | 0.723  (0.707 – 0.740) |
| F1 score | 0.566  (0.553 – 0.578) | 0.550  (0.534 – 0.566) | 0.559  (0.540– 0.578) | 0.677  (0.663 – 0.690) |


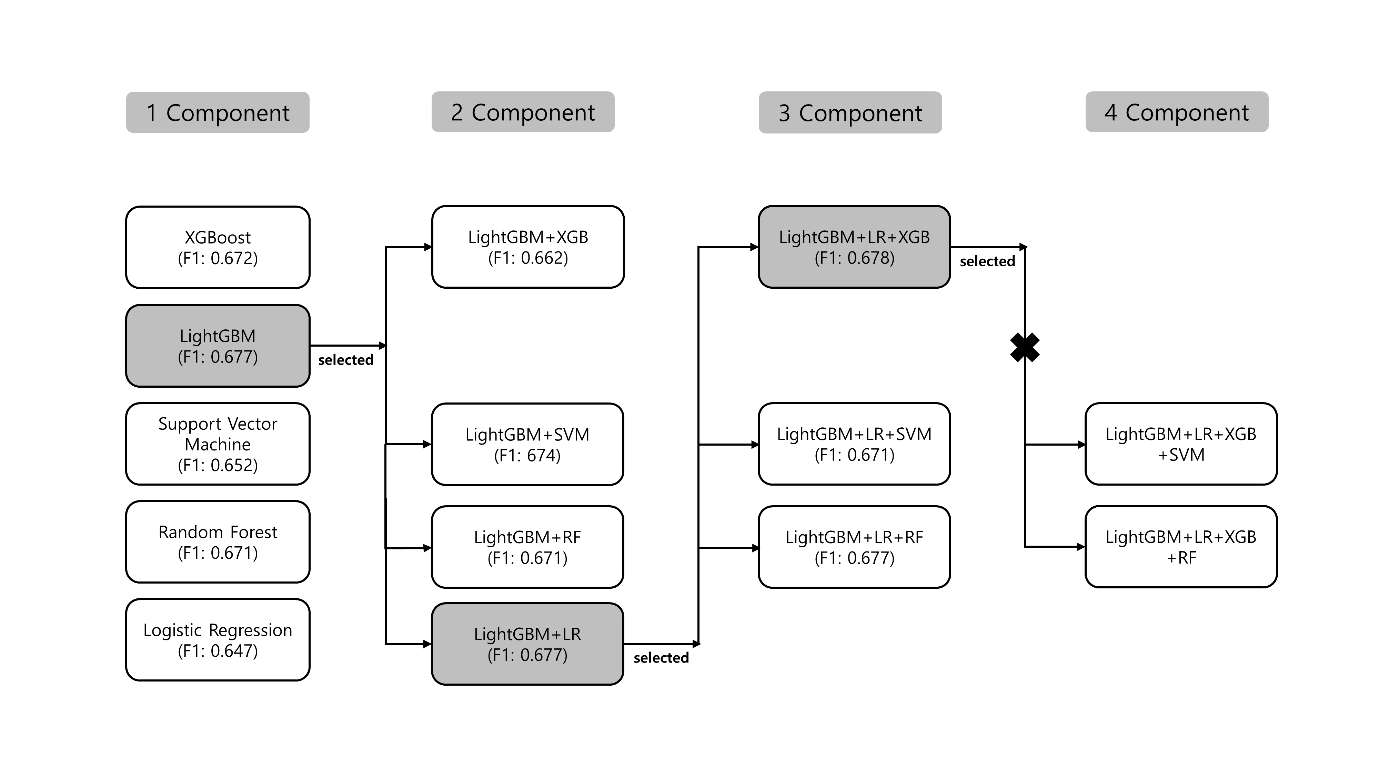


Supplementary Figure 1. Forward selection for the ensemble model of clinical features

LR, logistic regression; RF, random forest; SVM, support vector machine; XGB, XGBoost; F1, F1 score


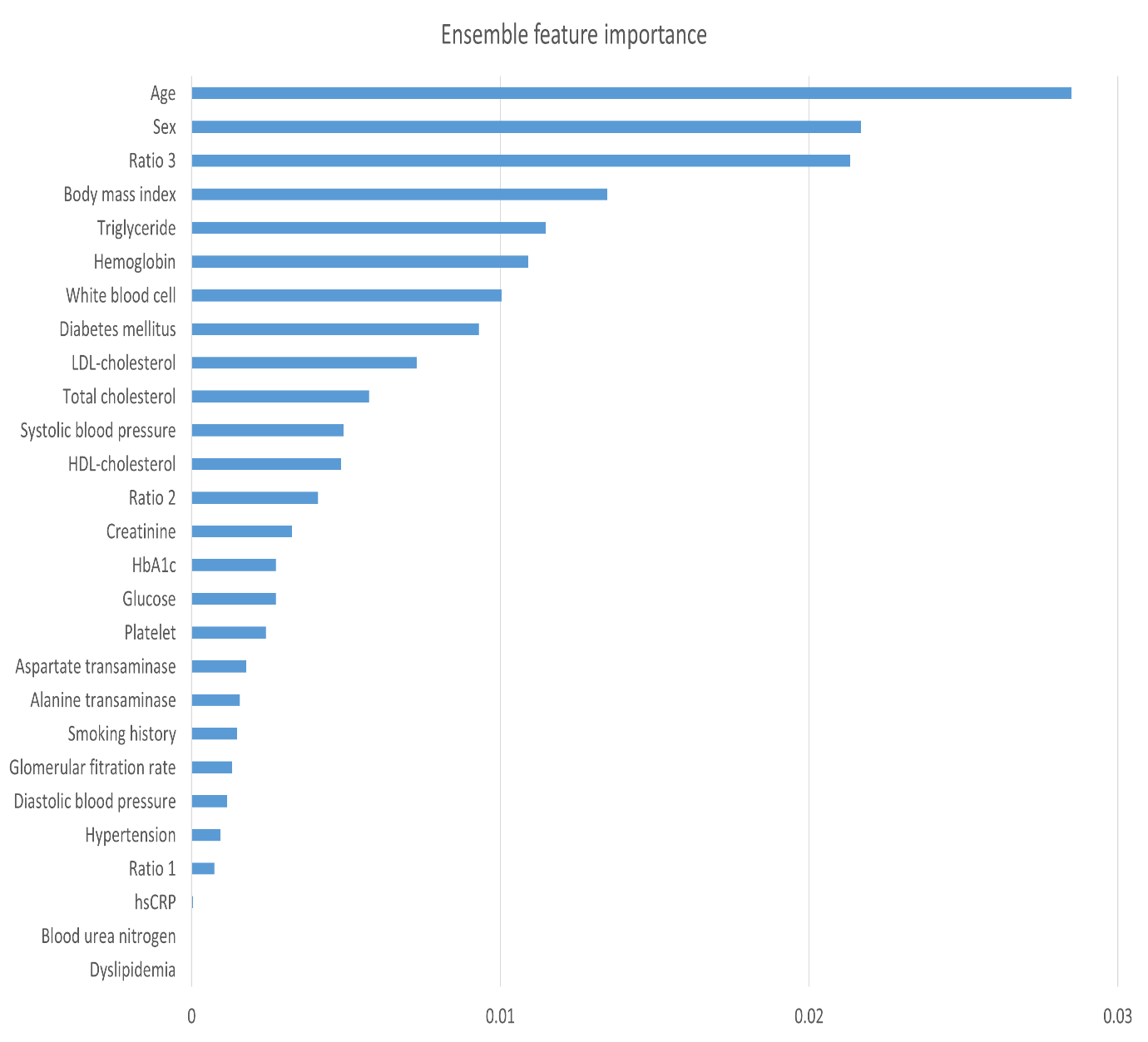


Supplementary Figure 2. Permutation feature importance of the clinical ensemble model

LDL, low density lipoprotein; HDL, high density lipoprotein; HbA1c, Hemoglobin A1c; hsCRP, high sensitivity C-reactive protein

Ratio 1: monocyte/HDL cholesterol, ratio 2: lymphocyte/monocyte, ratio 3: log(triglyceride/HDL-cholesterol)
